# Quantitative Assessment of Liver Steatosis and Affected Pathways with Molecular Imaging and Proteomic Profiling

**DOI:** 10.1038/s41598-018-22082-6

**Published:** 2018-02-26

**Authors:** Yasuyo Urasaki, Chi Zhang, Ji-Xin Cheng, Thuc T. Le

**Affiliations:** 1 Department of Biomedical Sciences, College of Medicine, Roseman University of Health Sciences, 10530 Discovery Drive, Las Vegas, NV 89135 USA; 2 Departments of Electrical and Computer Engineering & Biomedical Engineering, College of Engineering, Boston University, 8 St. Mary’s St, Boston, MA 02215 USA

## Abstract

Current assessment of non-alcoholic fatty liver disease (NAFLD) with histology is time-consuming, insensitive to early-stage detection, qualitative, and lacks information on etiology. This study explored alternative methods for fast and quantitative assessment of NAFLD with hyperspectral stimulated Raman scattering (SRS) microscopy and nanofluidic proteomics. Hyperspectral SRS microscopy quantitatively measured liver composition of protein, DNA, and lipid without labeling and sensitively detected early-stage steatosis in a few minutes. On the other hand, nanofluidic proteomics quantitatively measured perturbations to the post-translational modification (PTM) profiles of selective liver proteins to identify affected cellular signaling and metabolic pathways in a few hours. Perturbations to the PTM profiles of Akt, 4EBP1, BID, HMGCS2, FABP1, and FABP5 indicated abnormalities in multiple cellular processes including cell cycle regulation, PI3K/Akt/mTOR signaling cascade, autophagy, ketogenesis, and fatty acid transport. The integrative deployment of hyperspectral SRS microscopy and nanofluidic proteomics provided fast, sensitive, and quantitative assessment of liver steatosis and affected pathways that overcame the limitations of histology.

## Introduction

NAFLD affects nearly 30% of the general adult population^1^ and up to 70–80% of obese and diabetic populations worldwide^2^. NAFLD is characterized by a broad range of disorders from simple steatosis to non-alcoholic steatohepatitis (NASH)^3^. NASH is a common cause of end-stage liver disease such as cirrhosis and hepatocellular carcinoma, which require liver transplantation^4,5^. Due to the rising obesity epidemic and NAFLD incidence, NASH is projected to surpass hepatitis C viral infection and become the leading etiology among liver transplant patients in the United States within the next decade^6^. The prevalence of NAFLD highlights the urgent need to develop diagnostic and therapeutic strategies for this condition^7,8^.

Non-invasive diagnostics are currently the preferred clinical methods to assess NAFLD^9,10^. While practical and convenient, these methods are insensitive to the detection of NAFLD. For example, non-invasive imaging modalities such as ultrasonography, computed tomography, and magnetic resonance imaging are unable to discriminate microvesicular steatosis from macrovesicular steatosis, or detect fatty liver with less than 30% steatosis^11^. On the other hand, liver blood tests yield normal aminotransferase level in patients with hepatic steatosis^12^. Efforts to identify better non-invasive biomarkers to diagnose and define stages of NAFLD are ongoing^13^.

Histology of liver biopsies remains the gold standard for the diagnosis of NAFLD^14,15^. However, liver biopsy procedures can cause pain and discomfort and pose risks of complication to patients, thus, significantly limit their clinical utilization. A window of opportunity to study liver biopsies exists during the evaluation of donor livers prior to transplantation, where post-mortem collection of livers was performed^4^. With the rising prevalence of NAFLD worldwide, there is a general decline of healthy liver donors and an increasing need for NAFLD assessment in donor livers^16^. Unfortunately, histology analysis is time-consuming, which is not compatible with the need to minimize the duration of cold ischemia for donor livers. Furthermore, various histologic systems for qualitative assessment of NAFLD could lead to variable liver biopsy interpretation^17–19^. Hence, alternative methods that can quickly and quantitatively evaluate NAFLD in liver biopsies are highly desirable^20,21^.

In this study, normal and NASH liver biopsies were examined with novel molecular imaging and proteomic profiling technologies. Specifically, hyperspectral SRS microscopy and nanofluidic proteomics were deployed to measure liver steatosis and selective protein species, respectively. Hyperspectral SRS microscopy is a fast, quantitative, and label-free imaging method capable of resolving the composition of lipid, protein, and DNA in biological samples^22–25^. On the other hand, nanofluidic proteomics is an automated and multiplexed method that measures perturbations to specific protein species to identify affected signaling pathways or metabolic processes^26–29^. This study aims to demonstrate the capability of hyperspectral SRS microscopy and nanofluidic proteomics for rapid and quantitative assessment of liver steatosis and affected pathways, respectively.

## Results

### Quantitative assessment of liver steatosis with hyperspectral SRS microscopy

**Figure 1:**
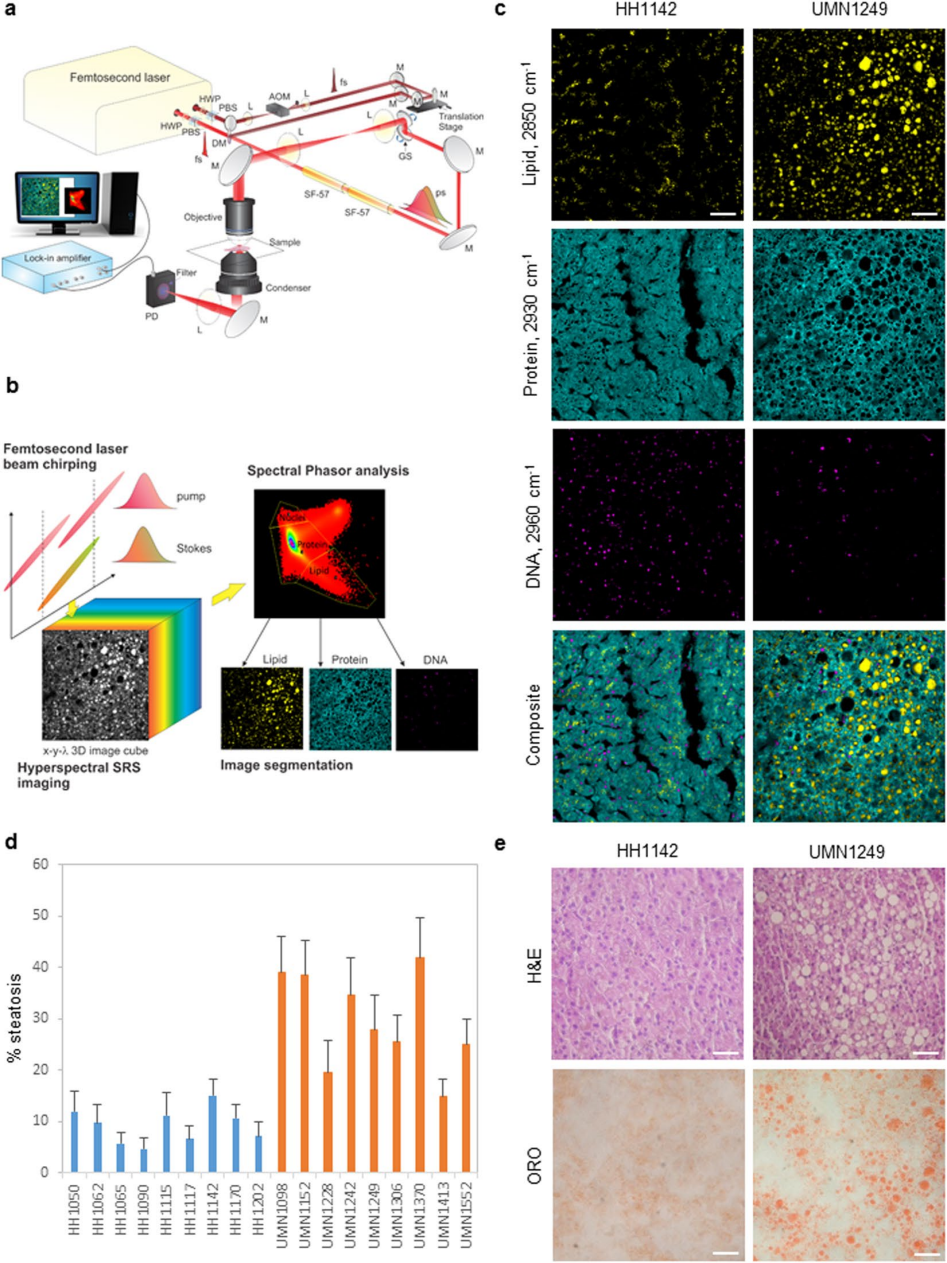
Imaging liver composition with hyperspectral stimulated Raman scattering microscopy. (**a**) Schematic of a hyperspectral SRS microscope. HWP: half-wave plate; PBS: polarizing beamsplitter; L: lens; AOM: acousto-optic modulator; fs: femtosecond; M: mirror; GS: galvanometer scanner; ps: picosecond; SF-57: glass block used to chirp pulses; PD: photodiode detector. (**b**) Schematic of a spectral-focusing-based hyperspectral SRS microscopy and spectral phasor segmentation of tissue compositions. (**c**) Decomposed images of lipid (2850 cm^−1^), protein (2930 cm^−1^), and DNA (2960 cm^−1^), and composite multicolor images of a normal liver (HH1142) and a NASH liver (UMN1249). Scale bars: 50 μm. (**d**) Percentage steatosis as a function of normal and NASH livers. Error bars are standard deviation across 27 stacked hyperspectral SRS images, or 1080 frames of 400 × 400 pixels/frame, used for spectral phasor analysis per liver sample. (**e**) H&E (upper panels) and ORO (lower panels) histology of HH1142 and UMN1249 liver specimen. Scale bars: 50 μm.

First, a home-built hyperspectral SRS microscope was deployed for label-free assessment of liver steatosis (Fig. 1a). Hyperspectral SRS imaging was performed using the spectral-focusing scheme outlined in Fig. 1b ^30^. To scan through the C-H vibration from 2800 cm^−1^ to 3050 cm^−1^, a mechanical optical delay stage in the Stokes beam was tuned at 10 microns per image, corresponding to a step of 5 cm^−1^. Each stacked hyperspectral SRS image was composed of 40 frames (400 × 400 pixels) acquired at 40 consecutive delay shifts with the acquisition time of 1.6 seconds per frame. Large-area imaging was achieved with stitching of stacked hyperspectral SRS images. A large-area hyperspectral SRS image of 1200 × 1200 pixels was acquired in approximately 10 minutes. Following image acquisition, spectral phasor analysis decomposed each stacked image into distinctive chemical compositions of lipid, protein, and DNA corresponding to different spectral clusters centering around 2850 cm^−1^, ~2930 cm^−1^, and 2960 cm^−1^, respectively (Fig. 1b,c & Supplemental Fig. S1)^31^. The SRS signals for lipid, protein, and DNA were used to assess liver steatosis, define the boundary of individual hepatocytes, and determine the location of the nuclei, respectively.

To quantitatively assess liver steatosis, the SRS signal of lipid as a function of the combined SRS signals of lipid, DNA, and protein was used to define the percentage of steatosis (Fig. 1d). As expected, NASH livers exhibited significantly higher percentage of steatosis compared to normal livers with an average of 28% versus 9%, respectively (Supplemental Tables S1 & S2). Interestingly, there was no statistically significant difference in the average number of lipid droplets detected in NASH livers versus normal livers. On the other hand, the average diameter of lipid droplets was two folds higher in NASH versus normal livers. All normal livers exhibited microvesicular steatosis with sub-micrometric lipid droplets. In contrast, all NASH livers exhibited macrovesicular steatosis with lipid droplets of highly variable diameters from sub-micrometer to 50 micrometers. Furthermore, less SRS DNA signals in the focal plane were detected in livers with macrovesicular steatosis compared to microvesicular steatosis (Fig. 1c), which could be attributed to the displacement of nuclei in the presence of large lipid droplets in macrovesicular steatosis. No statistically significant difference in SRS protein signal was detected between normal versus NASH livers. Clearly, hyperspectral SRS imaging was capable of rapid detection and quantitation of both liver microvesicular and macrovesicular steatosis in a label-free manner. By comparison, standard histology with hematoxylin and eosin (H&E) or oil red O (ORO) staining detect steatosis by the presence of vacuoles or red stains, respectively (Fig. 1e). While H&E and ORO histology were sufficient to detect macrovesicular steatosis in NASH livers (Fig. 1e, right panels), they were unable to detect microvesicular steatosis in normal livers (Fig. 1e, left panels).

### Quantitative assessment of affected liver pathways with nanofluidic proteomics

**Figure 2:**
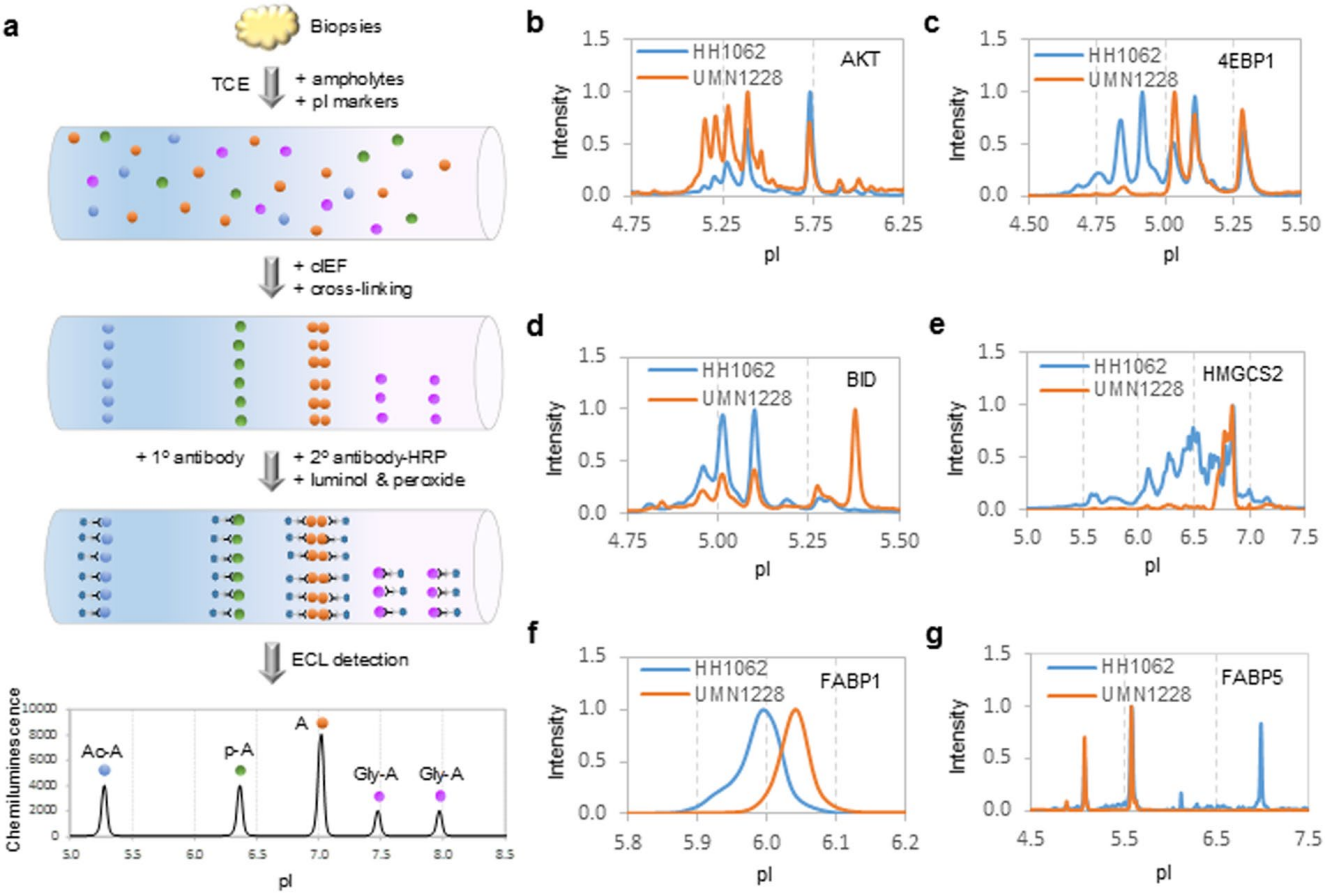
Profiling of selective liver protein species with nanofluidic proteomics. (**a**) Experimental flow of a typical IEF immunoassay to detect protein A species in a single capillary. TCE: total cell extract; pI: isoelectric point; cIEF: capillary isoelectric focusing; HRP: horseradish peroxidase; ECL: enhanced chemiluminescence; Ac-A: acetyl-isoforms; p-A: phosphor-isoforms; Gly-A: glycosyl-isoforms. (**b**–**g**) Representative cIEF electropherograms of (**b**) Akt, (**c**) 4EBP1, (**d**) BID, (**e**) HMGCS2, (**f**) FABP1, and (**g**) FABP5 in a control liver (HH1062, blue lines) versus a NASH liver (UMN1228, orange lines).

To complement the assessment of steatosis with hyperspectral SRS microscopy, nanofluidic proteomics was deployed to measure perturbations to protein species between normal and diseased liver tissues (Fig. 2a). Briefly, liver proteins were separated by charges via isoelectric focusing in capillaries. The locations of the proteins were stabilized with photo-cross-linking to the sidewalls of capillaries. Primary antibody to a specific protein of interest was introduced to each capillary, followed by the introduction of secondary antibody conjugated to horseradish peroxidase. Enhanced chemiluminescence was used to detect the presence of protein species within a single capillary. Protein species could be distinguished from one another based on shifts in pI values. For examples, acetyl-isoforms and phosphor-isoforms of a protein A cause shifts toward lower pI values compared to unmodified isoform, with acetyl-isoforms having much larger shifts than phosphor-isoforms^26^. In contrast, glycosyl-isoforms generally cause shifts toward higher pI values compared to unmodified isoform^26,32^. Multiplexed cIEF immunoassays were fully automated and capable of analyzing up to 96 different proteins in a single run. Out of more than forty liver proteins screened, six proteins were identified to have significant perturbations to protein species between normal and diseased states (Fig. 2b–g & Supplementary Fig. S2a–l). Representative electropherograms of a single normal (HH1062) versus diseased (UMN1228) liver samples were presented in Fig. 2b–g. Complete electropherograms of all nine normal and diseased liver samples were presented in Supplementary Fig. S2a–l. These proteins included protein kinase B (Akt), eukaryotic translation initiation factor 4E-binding protein 1 (4EBP1), BH3 interacting domain death agonist (BID), 3-hydroxy-3-methylglutaryl-CoA synthase 2 (HMGCS2), liver-specific fatty acid binding protein (FABP1), and epidermal fatty acid binding protein (FABP5).

**Figure 3:**
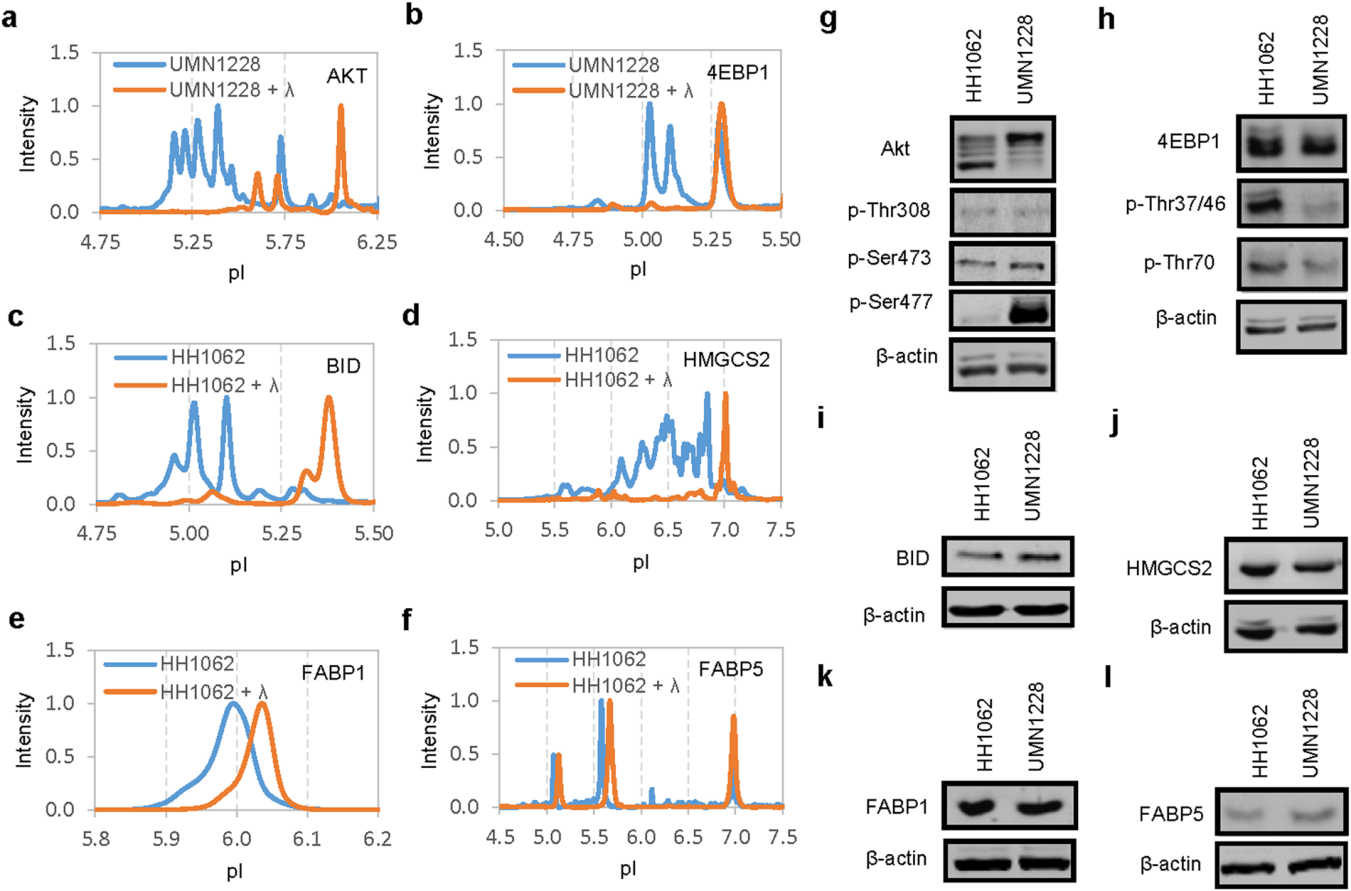
Evaluating protein phosphor-isoforms with cIEF immunoassays and Western blots. (**a–f**) Electropherograms of selective liver extracts before (blue lines) and after (orange lines) treatment with λ phosphatase for Akt (**a**), 4EBP1 (**b**), BID (**c**), HMGCS2 (**d**), FABP1 (**e**), and FABP5 (**f**). (**g–l**) Western blots of proteins of interest in a control liver (HH1062) versus a NASH liver (UMN1228) for (**g**) Akt, pAkt (Thr308), pAkt (Ser473), and pAkt (Ser477), (**h**) 4EBP1, 4EBP1 (p-Thr37/46), and 4EBP1 (p-Thr70), (**i**) BID, (**j**) HMGCS2, (**k**) FABP1, (**l**) FABP5. Cropped blots in **g** and **h** were of different gels that were loaded with the same amount of total cell extracts and probed with antibodies against Akt and 4EBP1 or their phosphor-isoforms. Cropped blots in **i**, **j**, **k**, and **l** were of the same gels. All gels were ran on the same day and subjected to the same experimental procedures. Immunoblots were detected with the same exposure duration. Membranes were stripped and re-incubated with antibodies against β-actin. Representative immunoblots of β-actin were presented to highlight comparable loading of total cell extracts between lanes.

To determine the contribution of phosphor-isoforms to the protein species, selective liver tissue extracts were treated with λ phosphatase and examined with cIEF immunoassays (Fig. 3a–f). Five proteins including Akt, 4EBP1, BID, HMGCS2 and FABP1 were highly sensitive to λ phosphatase treatment, which indicated the presence of significant phosphor-isoforms (Fig. 3a–e). On the other hand, FABP5 was not sensitive to λ phosphatase treatment (Fig. 3f). The mode of PTM to FABP5 was determined to be lysine acetylation (Supplementary Fig. S3). No significant glycosyl-isoform was detected with cIEF immunoassays for any of the six proteins analyzed. In addition, Western blots were performed on all nine normal and diseased liver samples for six proteins of interest. Representative Western blots of a single normal (HH1062) versus diseased (UMN1228) liver samples were presented in Fig. 3g–l. Western blots using antibodies specific to phosphor-isoforms of Akt and 4EBP1 identified increased phosphorylation of Akt at residue S477 and decreased phosphorylation of 4EBP1 at residues Thr37/46 and Thr70 in NASH versus normal livers (Fig. 3g & h). In contrast, Western blots of BID, HMGCS2, FABP1, and FABP4 without antibodies to specific phosphor-isoforms revealed no difference in their expression levels between NASH versus normal livers (Fig. 3i–l).

**Figure 4:**
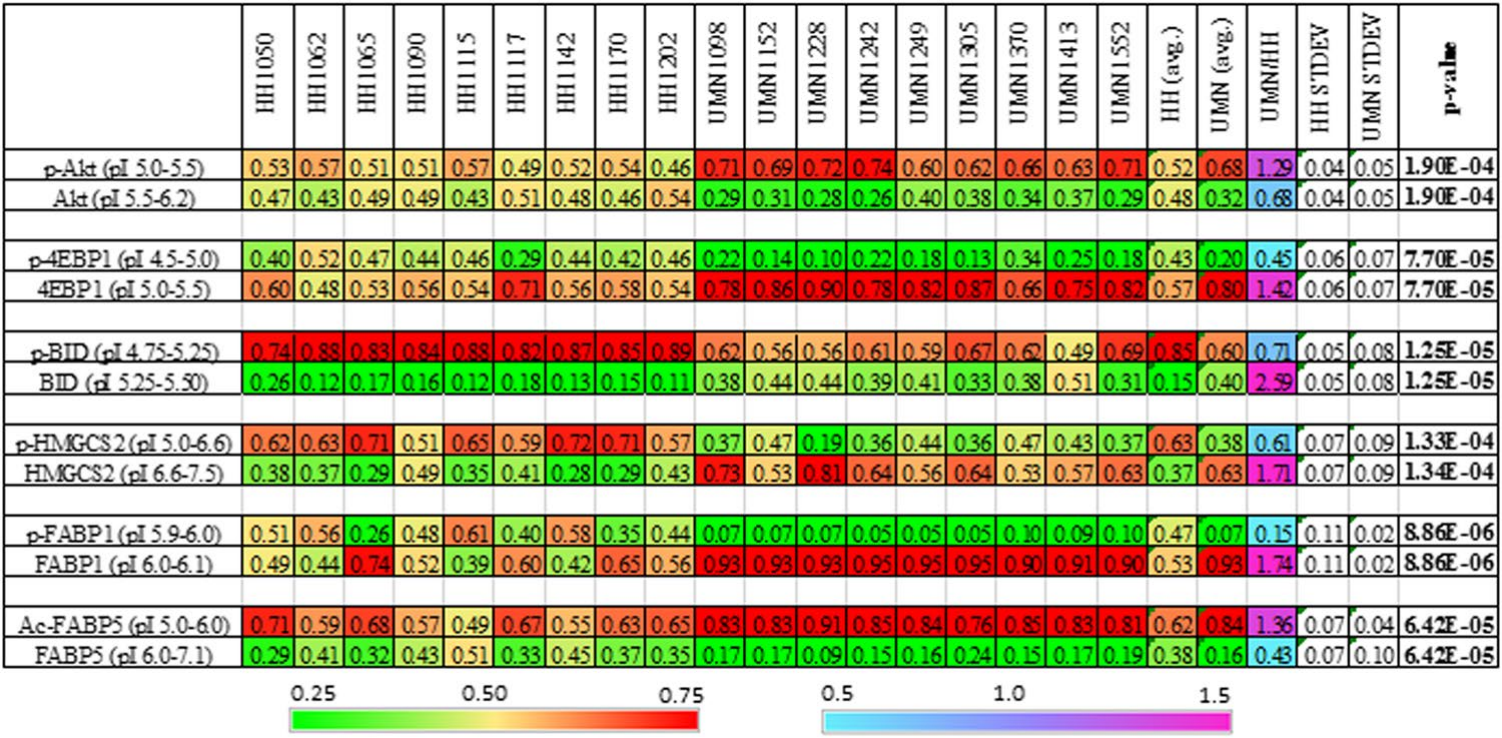
Relative concentrations of protein isoforms in normal and NASH livers. Two heat maps were generated for rapid assessment of the relative concentrations of protein species (left heat scale bar, green/yellow/red) and differences in the relative concentrations of protein isoforms between NASH and normal livers (UMN/HH, right heat scale bar, cyan/blue/red). Avg: average; STDEV: standard deviation. Statistical significance was set at p ≤ 0.05.

To quantitatively describe perturbations to protein species in NASH versus normal livers, the relative concentrations of individual protein species were calculated. The relative concentration of a protein species was defined as the ratio of its area under the curve (AUC) over total AUC of both modified and unmodified isoforms. The relative concentrations of selective protein species in normal and NASH livers were summarized in Fig. 4 with both numerical values and heat maps for rapid assessment of perturbations. Interestingly, phosphor-Akt isoforms were found to be significantly higher in NASH versus control livers. In contrast, phosphor-isoforms of 4EBP1, BID, HMGCS2, and FABP1 were consistently lower in NASH versus control livers. In addition, FABP5 was present mainly in acetyl-isoforms in NASH livers.

### Relationship between inhibition of cellular processes and perturbations to protein species

**Figure 5:**
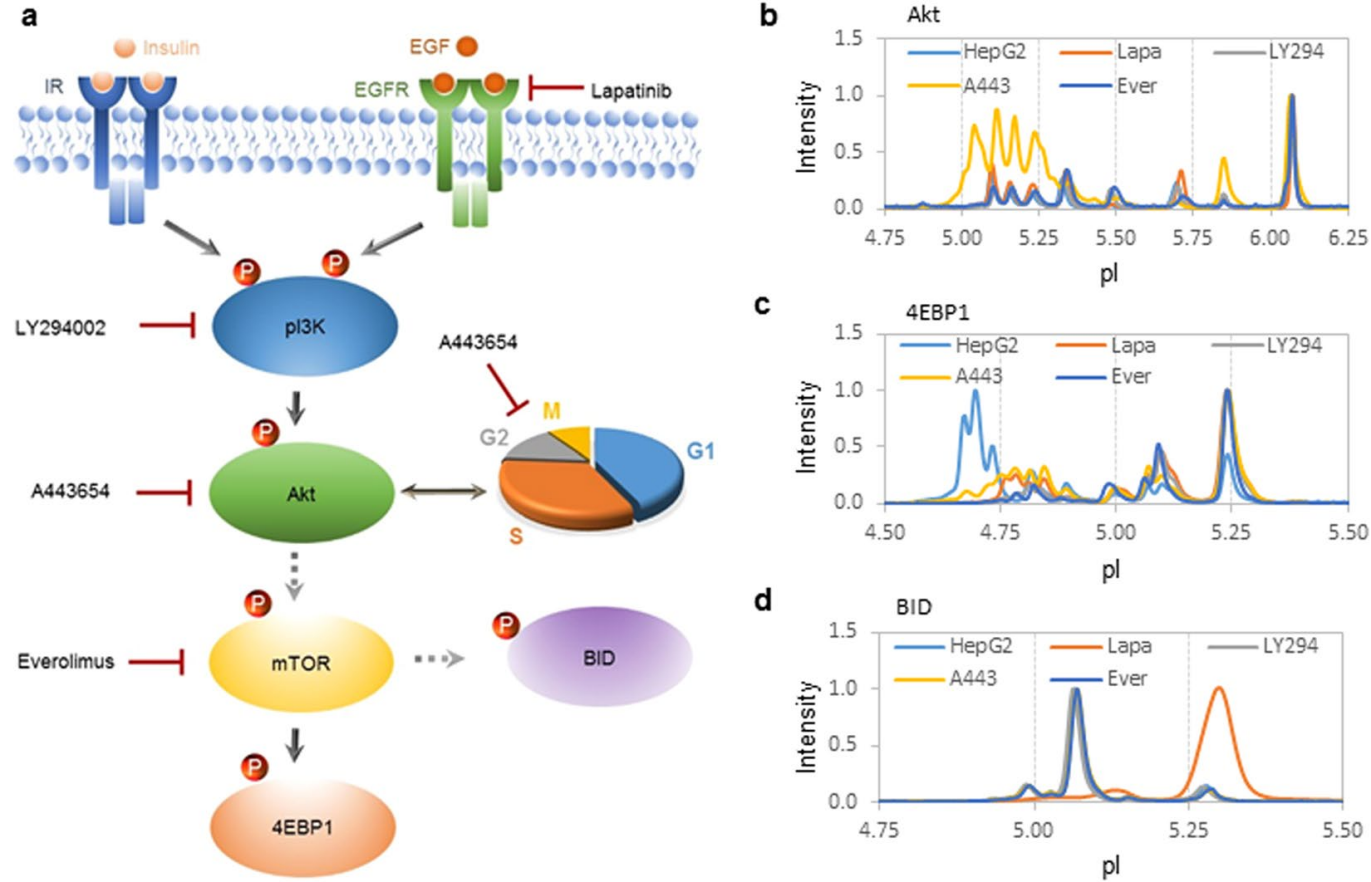
Relationship between inhibition of pI3K/Akt/mTOR signaling pathway and perturbations to protein species. (**a**) A diagram of pI3K/Akt/mTOR signaling pathway and its regulation of cell cycle progression, protein biosynthesis, and autophagy via the phosphorylation of Akt, 4EBP1, and BID, respectively. Targets of small-molecule kinase inhibitors are indicated with red blunted lines. Dashed arrows indicate indirect control via one or more intermediates. (**b–d**) Electropherograms of Akt (**b**), 4EBP1 (**c**), and BID (**d**) in HepG2 cells untreated (light blue line) or in treated with lapatinib (orange line), LY294002 (grey line), A443654 (yellow line), or everolimus (dark blue line).

The significance of perturbations to protein species in NASH livers was further examined in HepG2 cell cultures treated with various small-molecule kinase inhibitors targeting the PI3K/Akt/mTOR signaling pathway, which is an important regulator of cell cycle, protein biosynthesis, and autophagy^33^ (Fig. 5a). Treatment of HepG2 cells with inhibitors of EGFR (lapatinib), pI3K (LY294002), or mTOR (everolimus) had no observable effect on Akt phosphor-isoforms (Fig. 5b). Interestingly, treatment of HepG2 cells with an inhibitor of Akt (A443654) increased Akt phosphor-isoforms (Fig. 5b). On the other hand, treatment of HepG2 cells with lapatinib, LY294002, A443654, or everolimus all led to the suppression of 4EBP1 phosphor-isoforms (Fig. 5c). Treatment of HepG2 cells with lapatinib suppressed the expression of BID phosphor-isoforms (Fig. 5d) and induced the expression of an autophagy marker LC3A/B-II (Supplementary Fig. S4), whereas treatment of HepG2 cells with LY294002, A443654, or everolimus had no observable effect on the expression level of BID phosphor-isoforms. The effects of kinase inhibitors on HMGCS, FABP1, and FABP5 were also examined. However, HepG2 cells did not express HMGCS2^34^ and expressed FABP1 and FABP5 only in unmodified isoforms (Supplementary Fig. S5), which did not vary as a function of kinase inhibitor treatment.

## Discussion

This study demonstrated the capability of hyperspectral SRS microscopy and nanofluidic proteomics for fast and quantitative assessment of liver steatosis and affected pathways. In addition to morphologic assessment of steatosis resolved with label-free hyperspectral SRS imaging, nanofluidic proteomics data revealed that perturbations to protein species could serve as novel biomarkers to assess affected pathways in NAFLD. First, perturbation to a protein species could be used to assess its activity and function. For example, phosphorylation of HMGCS2 increases its enzymatic activity^35^. Suppression of HMGCS2 phosphor-isoforms in NASH livers could indicate impairment of ketogenesis and cholesterol biosynthesis. In addition, dephosphorylation or acetylation are mechanisms to inactivate fatty acid binding proteins^36,37^. Suppression of FABP1 phosphor-isoforms and elevation of FABP5 acetyl-isoforms in NASH livers could indicate impaired fatty acid transport. It is noteworthy to point out that NASH livers, but not control livers, expressed adipocyte- and macrophage-specific FABP4 (Supplementary Fig. S6), which is associated with lipid droplet accumulation^38^. Altered enzymatic activity and expression of selective fatty acid binding proteins could indicate a functional shift of hepatocytes from a lipid-processing function, which depends on FABP1 and FABP5 activities, to a lipid-storage function, which depends on FABP4 activity^39,40^.

Furthermore, perturbation to protein species could be used to assess cellular signaling activities. For example, interference with signaling activity of the PI3K/Akt/mTOR signaling pathway led to the suppression of 4EBP1 phosphor-isoforms in primary hepatocytes. By inference, suppression of 4EBP1 phosphor-isoforms could indicate impairment in PI3K/Akt/mTOR signaling pathway in NASH livers. On the other hand, inhibition of EGFR but not other proteins in the PI3K/Akt/mTOR signaling pathway led to the suppression BID phosphor-isoform and increased expression of an autophagy marker LC3A/B-II^41^. Suppression BID phosphor-isoform could potentially be used as a biomarker for liver autophagy signaling pathway that is independent of the PI3K/Akt/mTOR signaling pathway^42^.

In recent years, a mechanistic link between aberrant cell cycle progression in cancer and Akt hyper-phosphorylation at its carboxyl terminus has been discovered^43^. This study revealed that Akt hyper-phosphorylation at residue S477 was observed in NASH livers. Inhibition of Akt with A443654 also led to Akt hyper-phosphorylation at residue S477 in HepG2 cells (Supplementary Fig. S7). A443654 has been shown to interfere with mitotic progression and bipolar spindle formation^44^. Thus, Akt hyper-phosphorylation at residue S477 in NASH livers could indicate a dysregulation in the Akt-cell cycle pathway. NAFLD increases the risk of liver cancer although the underlying mechanisms have not been delineated^5^. Akt hyper-phosphorylation at residue S477 could potentially serve as a biomarker for the risk of progression of NAFLD to hepatocellular carcinoma although further investigation is warranted. Nanofluidic proteomics has been deployed for the detection of Akt phosphor-isoforms in various non-cancer and cancer tissues and could be instrumental for in-depth investigation of the roles of Akt phosphor-isoforms in fatty liver disease progression^29,45–48^. The assessment of inhibitor effects in HepG2 cells with nanofluidic proteomics suggested that multiple pathways were perturbed in NAFLD. It is plausible that perturbations to signaling pathways identified in this study could be used as biomarkers for the evaluation of liver function and drug treatment effects.

Most significantly, the integrative deployment of hyperspectral SRS microscopy and nanofluidic proteomics provided attractive alternatives to histology for the evaluation of NAFLD. Without the need for complex sample fixation, processing, or staining, label-free hyperspectral SRS imaging significantly reduced the assessment time for livers steatosis from several days to just a few minutes. Most importantly, hyperspectral SRS imaging was highly sensitive to the detection of microvesicular steatosis, which was not detectable with histology. In standard histology protocol, tissues were fixed in buffered formalin and embedded in paraffin for sectioning. Deparaffinization of tissue sections with a mixture of xylene and ethanol also removed lipid droplets with sub-micrometric diameters^49^. Consequently, the assessment of liver steatosis with histology either failed to detect or underestimated microvesicular steatosis^21^. Microvesicular steatosis is a liver lesion that can be associated with liver failure, encephalopathy, and hypoglycemia with fatal consequences in some patients^50^. Hepatic microvesicular steatosis is observed in Rey’s syndrome, acute fatty liver of pregnancy, or acute liver injury due to drug-induced mitochondrial and metabolic toxicity^50,51^. Hyperspectral SRS imaging of hepatic microvesicular steatosis provided a unique capability for the detection of early-stage steatosis for the assessment of liver health. Additionally, quantitative analysis of hyperspectral SRS imaging data eliminated common errors associated with traditional qualitative steatosis assessment of histological data^18^.

On the other hand, nanofluidic proteomics quantitatively measured perturbations to the PTM profiles of selective proteins to identify abnormalities in associated cellular processes. Significantly, nanofluidic proteomics allowed analysis of protein species using less than 40 ng of total cellular protein per assay^28,52,53^. By comparison, Western blot analysis required approximately 10 µg of total cellular protein per assay^52^. Using less than the amount of total cellular protein required for a single Western blot, up to 96 cIEF immunoassays could be performed in a single run with the timescale of several hours^54^. The utilization of nanograms-size samples rendered nanofluidic proteomics highly suitable for multiplexed analysis of protein species in finite liver tissue biopsies. Interestingly, perturbations to liver protein species could serve as diagnostic biomarkers for NAFLD stages (Table 1). For example, previous profiling of liver protein PTM revealed that inactivation of FABP1 and FABP5 by acetylation and dephosphorylation, respectively, expression of FABP4, and dephosphorylation of 4EBP1, HMGCS2, and Akt at residue S473 were observed in animal models with simple macrovesicular steatosis^26,32^. This study further revealed that dephosphorylation of BID and hyper-phosphorylation of Akt at residue S477 were associated with human NASH liver tissues. Thus, the impairment of fatty acid transport, insulin signaling pathway, ketogenesis and cholesterol biosynthesis were likely early events in NAFLD development. In contrast, activation of autophagy and impaired cell cycle regulation were likely late events in NAFLD development. Future development of an expanded pathway-focused diagnostic panel of protein species should permit the evaluation of causes, consequences, and risks of progression for NAFLD with nanofluidic proteomics^55^. Together, the integrative deployment of hyperspectral SRS microscopy and nanofluidic proteomics provided fast and quantitative assessment of steatosis and affected pathways in NAFLD that overcame the limitations of traditional histology. Hyperspectral SRS microscopy and nanofluidic proteomics are highly suitable for the time-sensitive evaluation of liver health such as during the assessment of donor livers for transplantation or surgeries.

**Table 1:** Perturbations to protein species as biomarkers of NAFLD stages.

Perturbations	Affected pathways	Early-stage NAFLD (Macrovesicular steatosis)	Late-stage NAFLD (NASH)
FABP1 dephosphorylation or acetylation	Fatty acid transport	✓	✓
FABP4 expression	Fatty acid transport	✓	✓
FABP5 dephosphorylation or acetylation	Fatty acid transport	✓	✓
4EBP1 dephosphorylation	Insulin signaling	✓	✓
HMGCS2 dephosphorylation	Ketogenesis & cholesterol biosynthesis	✓	✓
BID dephosphorylation	Autophagy		✓
Akt dephosphorylation at S473	Insulin signaling	✓	
Akt hyperphosphorylation at S477	Cell cycle regulation		✓

## Methods

### Human liver biopsies

Frozen human liver biopsies classified as normal (n = 9) or with NASH (n = 9) were provided by the Liver Tissue Cell Distribution System (LTCDS, Minneapolis, Minnesota), which was funded by the National Institutes of Health Contract # HSN276201200017C (Supplementary Table S1). This study used collected specimen that had been de-identified by the LTCDS and was exempted by the Roseman University of Health Sciences Institutional Review Board (protocol # 16-SM-MD-0701).

### Hyperspectral SRS microscopy

The hyperspectral SRS microscope system was built on an upright microscope frame (Olympus BX51) using the InSight DS^+^ ultrafast laser system (Spectra-Physics, Santa Clara, CA). The laser system provided a Stokes beam at 1040 nm (120 femtoseconds) and a pump beam tunable from 690–1300 nm (150 femtoseconds) with a repetition rate of ~80 MHz. The Stokes beam was modulated at ~2.5 MHz by a function generator. The pump and Stokes beams were combined by a dichroic mirror and chirped to picosecond pulses by two 30 cm SF-57 glass rods (Lattice Electro Optics, Fullerton, CA). A 2D galvo system (GVSM002, Thorlabs, Newton, NJ) was used for laser scanning and a 40 × water-immersion objective lens (LUMPlanFLN × 40, Olympus, Waltham, Massachusetts) was used to focus the laser beams into the sample. An oil condenser (NA = 1.4) was used to collect forward-scattered light into a photodiode detector (S3994–01, Hamamatsu, Bridgewater, NJ). A short-pass filter (980 SP, Chroma, Bellows Falls, VT) was used to reject the Stokes beam from entering the photodetector. The signal was deconvoluted and amplified by a lock-in amplifier (HF2LI, Zurich Instrument, Zurich, Switzerland). Large-area mapping was performed by synchronizing the motorized 2D linear stage to the hyperspectral image collection. To avoid spectral distortion, 40 spectral frames (400 × 400 pixels for each frame) were collected before the stage was moved to an adjacent area. LabView software was used for image collection, stitching, and display. Liver lipid droplet size and number was analyzed with ImageJ as described previously^21^.

### Spectral phasor analysis

Stacked hyperspectral images were imported to ImageJ with phasor analysis plugin. Phasor transformation segmented chemical compositions to different clusters. Gating for different clusters was manually selected and kept identical across images. Segmentation images were plotted using the ‘Phasor to image’ command over different gates selected in the phasor domain.

### Preparation of liver tissues for imaging

Frozen liver tissues were embedded in optimal cutting temperature compound, flash-frozen in liquid nitrogen, sectioned into 20-micron slices with a cryotome, and sandwiched between two coverslips for hyperspectral SRS imaging. On average, three tissue sections per liver sample were used for hyperspectral SRS imaging. For each tissue section, hyperspectral SRS images of at least nine frames of 400 × 400 pixels per frame were acquired.

### Histology

Liver tissues were also used for independent histological analysis of liver steatosis with hematoxylin and eosin (H&E) or Oil Red O (ORO) stains by IHCWorld, LLC (Woodstock, MD).

### Preparation of liver tissue lysates

Approximately 50 mg of frozen liver tissues was added to 300 µl of Bicine/CHAPS Lysis Buffer (Cat. No. 040–764, Protein Simple, Santa Clara, CA) containing proteinase and phosphatase inhibitors and homogenized twice at 6 seconds duration. Liver tissue homogenates were incubated on ice for 10 minutes, sonicated 4 times at 5 seconds duration, rotated at 4 °C for 2 hours, and centrifuged at 12000 rpm on an Eppendorf 5430 R microfuge for 20 minutes at 4 °C. Supernatant was collected, prepared in Premix G2 pH 5–8 separation gradient containing pI standards (ProteinSimple), and used for capillary isoelectric focusing (cIEF) immunoassays.

### Treatment with phosphatase

Approximately 1 µl of 𝜆 phosphatase (Cat. No. 14–405, Merck Millipore, Billerica, MA) was added to 1 µl of reaction buffer (final concentrations of 5 mM DDT, 50 mM Hepes, 100 µM EDTA, 2 mM MnCl_2_) and 8 µl of liver tissue lysates (2 mg/ml of total protein concentration). The mixture was incubated at 37 °C for 30 minutes, chilled on ice to stop reaction, prepared in Premix G2 pH 5–8 separation gradient containing pI standards (ProteinSimple), and used for cIEF immunoassays.

### Multiplexed cIEF immunoassays

Multiplexed cIEF immunoassays were performed using the NanoPro 1000 system (Protein Simple). Samples of 400-nanoliter volume were separated by isoelectric focusing using the 96-capillary system and followed by immobilization of the proteins onto the inner capillary walls with ultraviolet irradiation. Primary antibodies (Supplemental Table S3) and horseradish peroxidase-conjugated secondary antibodies (Cat. No. 7074, Cell Signaling) were sequentially introduced into the capillaries and followed by chemiluminescence detection reagents. The incubation times were 110 and 55 minutes for primary and secondary antibodies, respectively. Separation time was 50 minutes at 15,000 MicroWatts. On average, 40 ng of total cellular protein was loaded into each capillary. Standard exposure time during signal detection was 240 seconds. A minimum of four repeats were done for each protein per liver sample. High fidelity between repeated measurements was consistent with published reports with coefficient of variation values of ≤ 0.1^54^.

### Analysis of cIEF immunoassay data

Peak intensities of all cIEF immunoassay electropherograms were normalized to 1. The relative concentration of a specific protein species was calculated as the ratio of area under the curve (AUC) for the isoform over total AUC of all isoforms.

### Statistical analysis

Statistical analysis was performed using a two-tailed paired Student’s t-test for NASH livers versus normal livers. Statistical significance was set at p ≤ 0.05.

### Western blots

Total liver protein extracts were separated on 10% SDS-PAGE gels, transferred to nitrocellulose membranes, incubated first with primary antibodies against proteins or protein phosphor-isoforms of interest (Supplementary Table S3) and then with IRDye 680RD secondary antibodies (Cat. No. 92668070, LI-COR, Lincoln, NE). Immunoblots were detected with the LI-COR’s Odyssey CLx imaging system. Membranes were stripped and re-incubated with antibodies against β-actin, which served as a loading control.

### HepG2 cell cultures

HepG2 cells were cultured in RPMI media (Cat. No. 11875–093, Gibco, Gaithersburg, MD) supplemented with 10% FBS (Cat. No. SH30070.03, Hyclone, Logan, Utah), non-essential amino acids (Cat. No. 25-025-CI, Corning, NY) and antibiotics penicillin and streptomycin (Cat. No. 15140, Gibco). For cells receiving treatment, small-molecule kinase inhibitors were added for 24 hours prior to analysis. The following kinase inhibitors were used at indicated final concentrations: lapatinib (5 µM, Cat. No. S2111 Selleckchem, Houston, TX), LYS294002 (5 µM, CalBioChem, Temecula, CA), A-443654 (0.5 µM, Cat. No. 16499, Cayman Chemical, Ann Arbor, MI), and everolimus (10 µM, Cat. No. S1120, Selleckchem). Concentrations of kinase inhibitors were selected based on our own screens of the effective concentrations to achieve maximal hyper-phosphorylation of Akt or dephosphorylation of 4EBP1.

### Data availability

The authors declare that data supporting the findings of this study are available within the paper and its supplementary information files.

## Electronic supplementary material

Supplementary Information
