# Supplementary material for: Quantitative Assessment of Liver Steatosis and Affected Pathways with Molecular Imaging and Proteomic Profiling
Source: Sci Rep. 2018 Feb 26;8:3606. doi: 10.1038/s41598-018-22082-6 (PMC5826939; doi:10.1038/s41598-018-22082-6)
Supplement: Supplementary file 1 — Supplementary Information [file 41598_2018_22082_MOESM1_ESM.pdf]

Supplementary Information

Quantitative Assessment of Liver Steatosis and Affected Pathways with Molecular Imaging and Proteomic Profiling

Yasuyo Urasaki[1, a], Chi Zhang[2, a], Ji-Xin Cheng[2*] & Thuc T. Le[1]*

[1]Department of Biomedical Sciences, College of Medicine, Roseman University of Health Sciences, 10530 Discovery Drive, Las Vegas, NV 89135, USA

[2]Departments of Electrical and Computer Engineering & Biomedical Engineering, College of Engineering, Boston University, 8 St. Mary's St, Boston, MA 02215, USA

[a]Equal contribution

*Corresponding authors

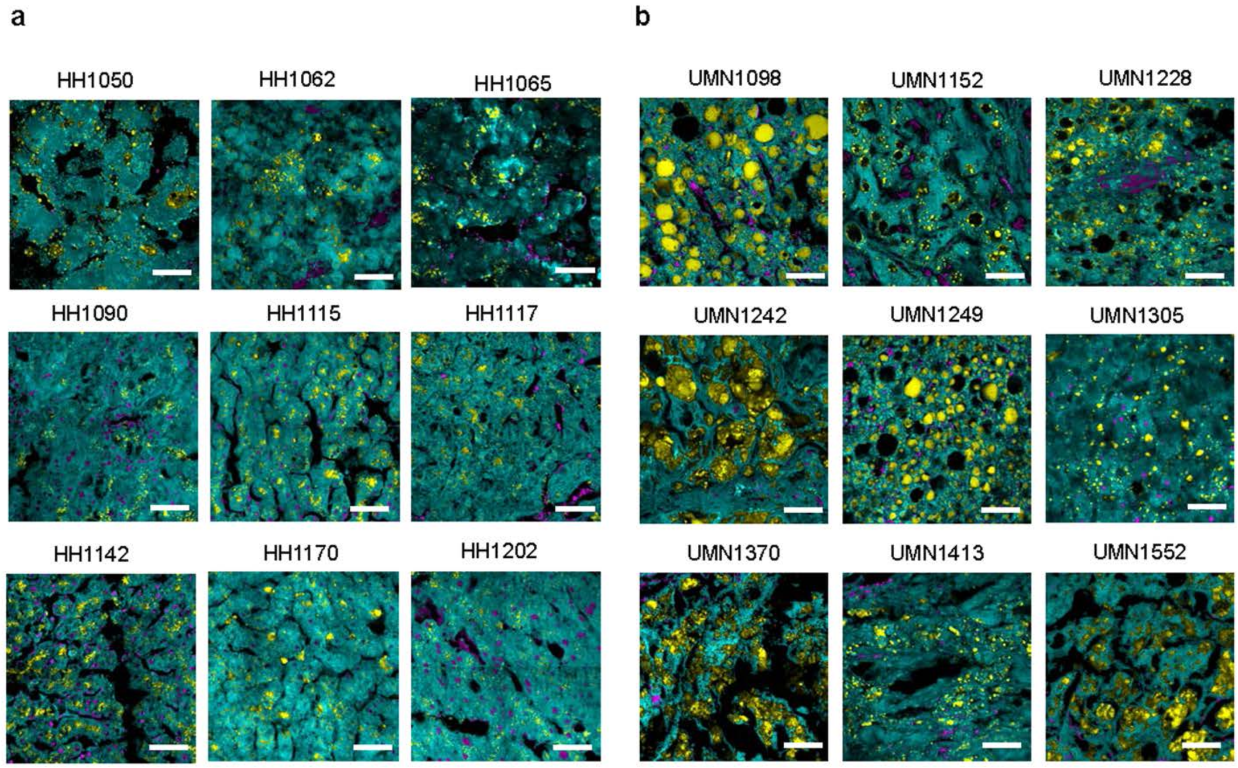

Supplementary Figure S1. Selective composite hyperspectral SRS images of normal (**a**) and NASH (**b**) livers. Lipid (yellow), protein (blue), and DNA (magenta). Scale bars: 50 µm.

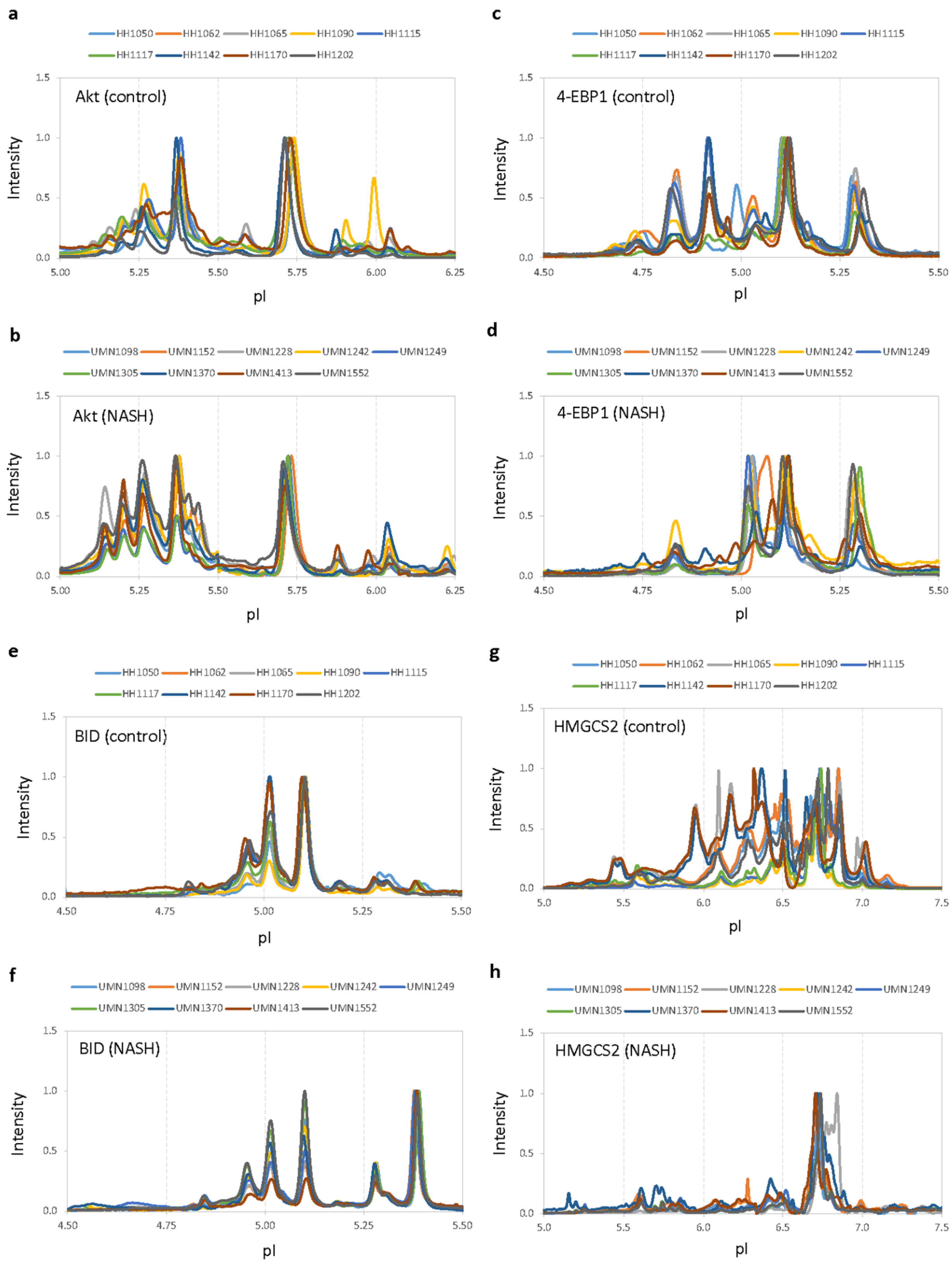
a
HH1050 HH1062 HH1065 HH1090 HH1115
HH1117 HH1142 HH1170 HH1202
Akt (control)
Intensity
pI
c
HH1050 HH1062 HH1065 HH1090 HH1115
HH1117 HH1142 HH1170 HH1202
4-EBP1 (control)
Intensity
pI
b
UMN1098 UMN1152 UMN1228 UMN1242 UMN1249
UMN1305 UMN1370 UMN1413 UMN1552
Akt (NASH)
Intensity
pI
d
UMN1098 UMN1152 UMN1228 UMN1242 UMN1249
UMN1305 UMN1370 UMN1413 UMN1552
4-EBP1 (NASH)
Intensity
pI
e
HH1050 HH1062 HH1065 HH1090 HH1115
HH1117 HH1142 HH1170 HH1202
BID (control)
Intensity
pI
g
HH1050 HH1062 HH1065 HH1090 HH1115
HH1117 HH1142 HH1170 HH1202
HMGCS2 (control)
Intensity
pI
f
UMN1098 UMN1152 UMN1228 UMN1242 UMN1249
UMN1305 UMN1370 UMN1413 UMN1552
BID (NASH)
Intensity
pI
h
UMN1098 UMN1152 UMN1228 UMN1242 UMN1249
UMN1305 UMN1370 UMN1413 UMN1552
HMGCS2 (NASH)
Intensity
pI

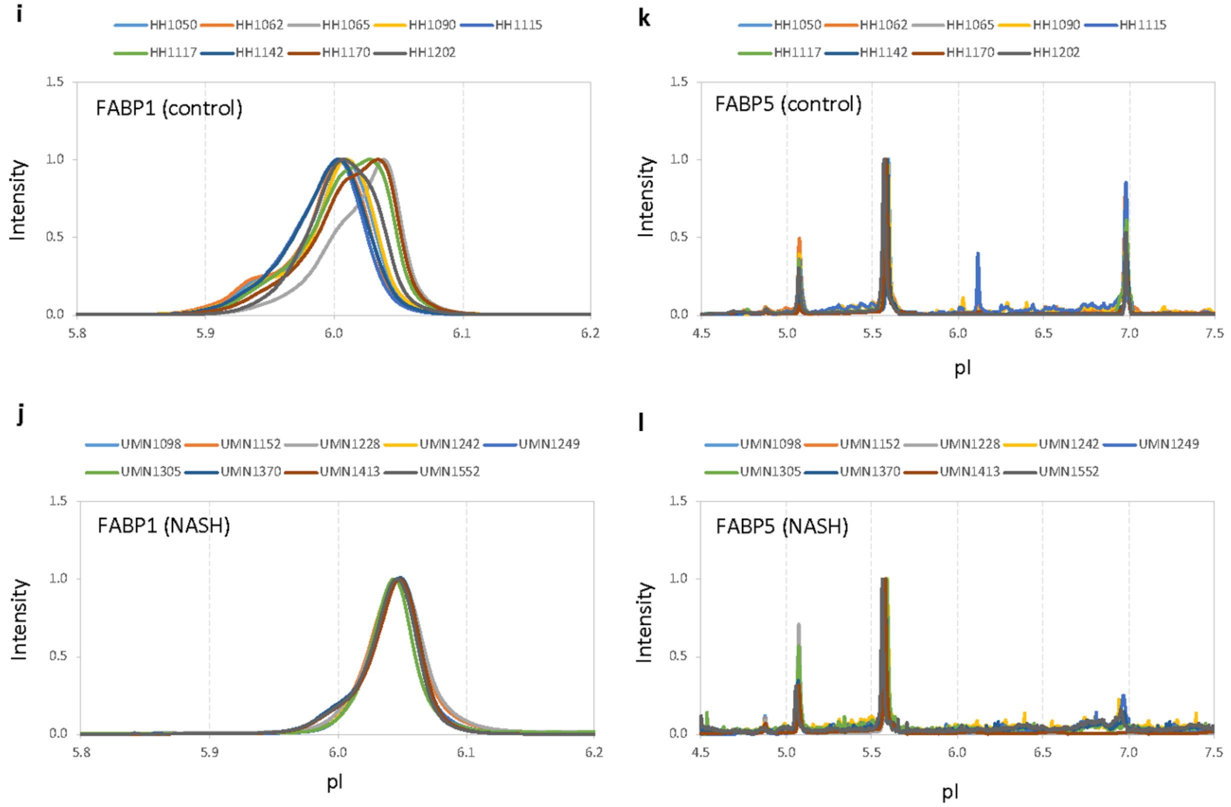

Supplementary Figure S2. Electropherograms of selective proteins in normal and NASH livers. Electropherograms of Akt in normal (**a**) and NASH (**b**) livers, 4EBP1 in normal (**c**) and NASH (**d**) livers, BID in normal (**e**) and NASH (**f**) livers, HMGCS2 in normal (**g**) and NASH (**h**) livers, FABP1 in normal (**i**) and NASH (**j**) livers, and FABP5 in normal (**k**) and NASH (**l**) livers.

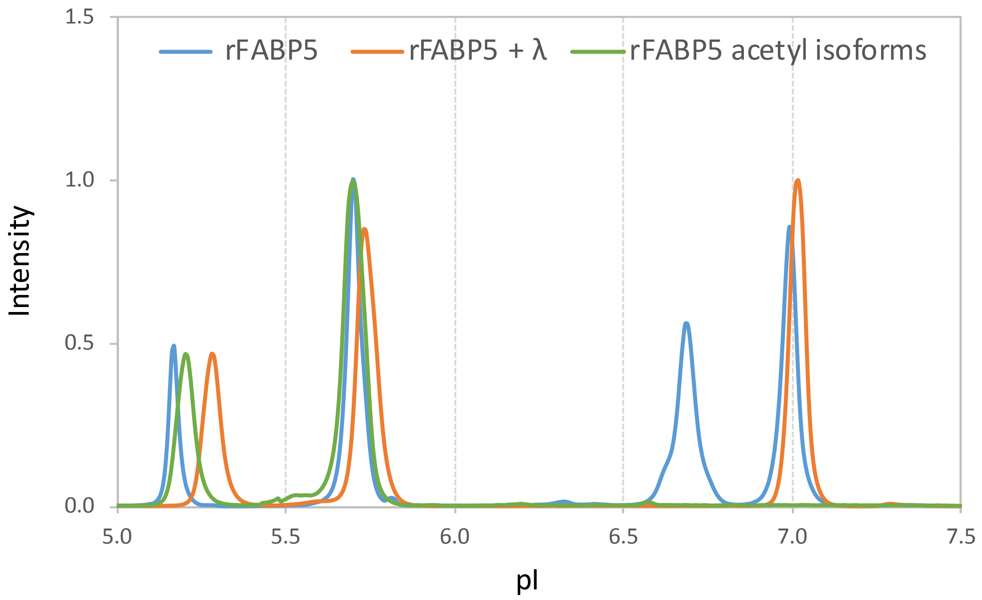

Supplementary Figure S3. Identifying FABP5 protein species on electropherograms. When probed with antibodies against FABP5, purified human recombinant FABP5 (rFABP5, Cat. No. TP301973, Origene, Rockville, MD) exhibited four distinctive peaks at pI 7.0, pI 6.7, pI 5.7, and pI 5.2 (blue line). Following the treatment of rFABP5 with λ phosphatase, only the rFABP5 peak at pI 6.7 was removed (orange line). On the other hand, when probed with antibodies directed against acetylated lysine residue (Cat. No. CS9441, Cell Signaling, Danvers, MA), two distinctive peaks at pI 5.2 and 5.7 were detected (green line). Experimental data supported the assignment of the following pI values to FABP5 isoforms: pI 6.9-7.2: unmodified FABP5, pI 6.2 – 6.9: FABP5 phosphor-isoforms, and pI 5.0-6.0: FABP5 acetyl-isoforms.

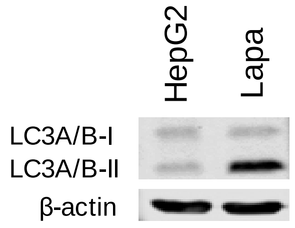

Supplementary Figure S4. Increased expression level of LC3A/B-II in HepG2 cells treated with EGFR inhibitor lapatinib. β-actin served as a loading control.

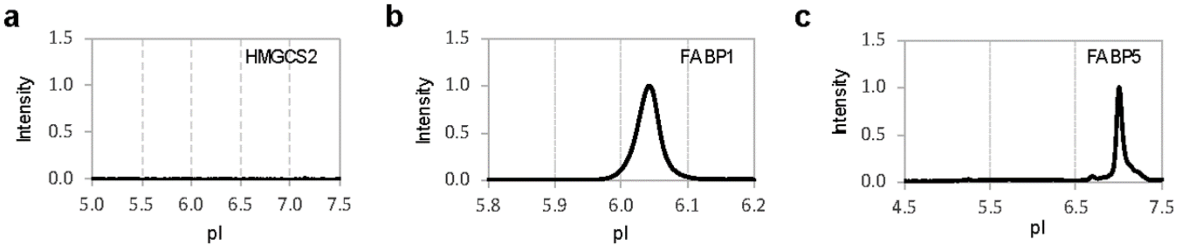

Supplementary Figure S5. Expression of HMGCS2, FABP1, and FABP5 in HepG2 cells. (**a**) Expression of HMGCS2 was undetectable in HepG2 cells. (**b**) FABP1 and (**c**) FABP5 proteins were expressed as unmodified isoforms in HepG2 cells.

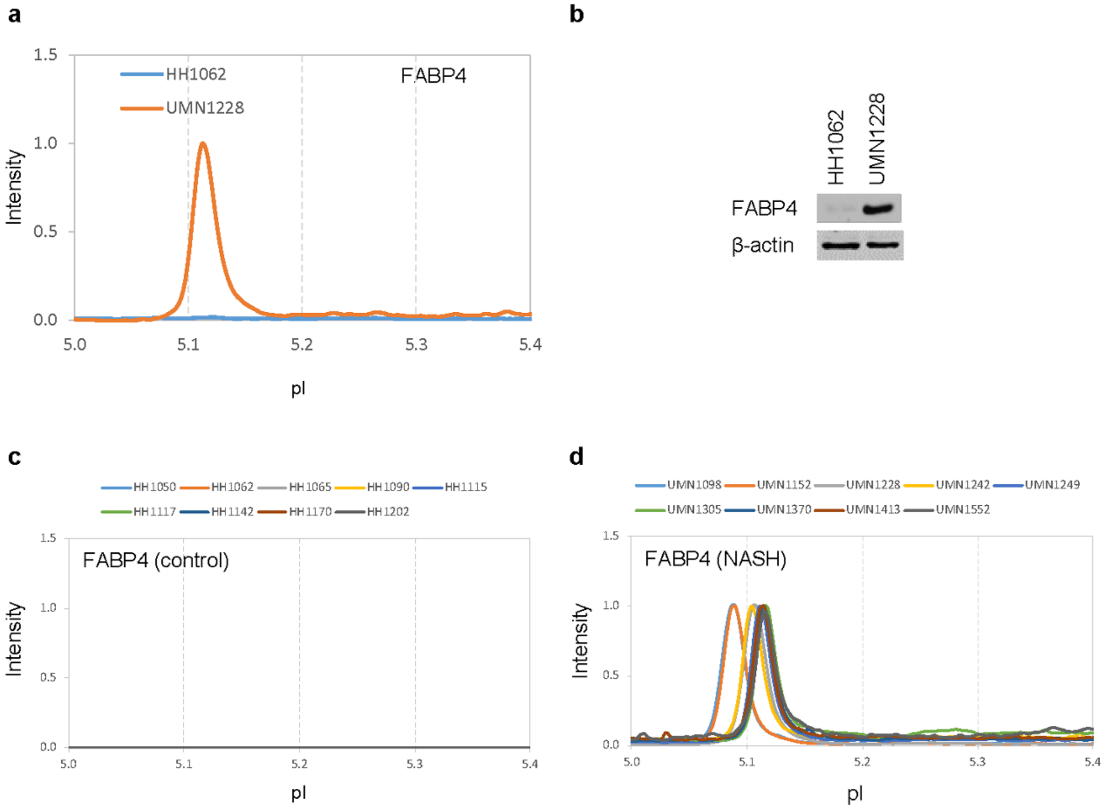

Supplementary Figure S6. Expression of FABP4 in NASH livers. (**a**) cIEF immunoassay electropherograms of FABP4 in normal liver (HH1062) and NASH liver (UMN1228). (**b**) A Western blot analysis of FABP4 expression level in HH1062 and UMN1228 livers. β-actin served as loading controls. (**c**) cIEF immunoassay electropherograms of FABP4 in 9 normal livers. (**d**) cIEF immunoassay electropherograms of FABP4 in 9 NASH livers.

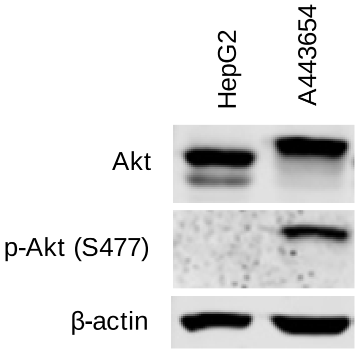

Supplementary Figure S7. Increased expression level of p-Akt (S477) in HepG2 cells treated with A443654. β-actin served as a loading control.

Supplementary Table S1. List of human liver specimen

ID Nº	Diagnosis	Sex	Age	COD
HH1050	Normal	M	58	HT
HH1062	Normal	M	39	CVA
HH1065	Normal	M	18	GSW/head
HH1090	Normal	F	28	ICH
HH1115	Normal	M	22	HT/MVA
HH1117	Normal	F	68	ICB
HH1142	Normal	M	19	HT/MVA
HH1170	Normal	F	49	ICH
HH1202	Normal	F	67	CVA
UMN1098	NASH	F	58	N/A
UMN1152	NASH	F	40	N/A
UMN1228	NASH	M	62	N/A
UMN1242	NASH	M	48	N/A
UMN1249	NASH	F	55	N/A
UMN1305	NASH	F	48	N/A
UMN1370	NASH	M	53	N/A
UMN1413	NASH	F	62	N/A
UMN1552	NASH	F	43	N/A

COD: cause of death; HT: head trauma; CVA: Cerebrovascular accident; GSW: gunshot wound; ICH: intracerebral hemorrhage; HT: head trauma; MVA: motor vehicle accident; ICB: intracranial bleeding; NASH: non-alcoholic steatoheptatitis; M: male; F: female; N/A: not available. Normal liver specimen were collected from deceased individuals with various COD. NASH liver specimen were collected from patients who received liver transplants. Information on human liver specimen was provided by the Liver Tissue Cell Distribution System (LTCDS, Minneapolis, Minnesota), which was funded by the National Institutes of Health Contract # HSN276201200017C.

Supplementary Table S2. Quantitative analysis of liver steatosis

	% steatosis	LD size (µm, diameter)	LD number
HH1050	11.95	0.55	2193
HH1062	9.71	0.68	1230
HH1065	5.69	5.69	892
HH1090	4.70	0.55	786
HH1115	11.25	0.71	2836
HH1117	6.69	0.63	1077
HH1142	15.05	0.92	2376
HH1170	10.65	0.66	1359
HH1202	7.11	0.59	3672
UMN1098	39.03	1.36	1276
UMN1152	38.43	0.61	2064
UMN1228	19.62	0.88	2033
UMN1242	34.74	2.76	385
UMN1249	27.82	1.14	3240
UMN1306	25.45	1.16	2573
UMN1370	42.01	1.95	593
UMN1413	14.91	0.67	1206
UMN1552	24.94	1.71	763
HH (avg.)	**9.20**	**0.65**	**1825**
UMN(avg.)	**27.77**	**1.36**	**1570**
UMN/HH	3.02	2.09	0.86
HH STDEV	3.38	0.12	997
UMN STDEV	8.23	0.69	967
p-value	0.00	0.02	0.61

Avg: average; STDEV: standard deviation. Analyses of percentagae steatosis were done with spectral phasor analysis and analyses of lipid droplet size and number were done with particle tracking function using the ImageJ software. All analysese were done on three large-area stacked hyperspectral SRS images with each image consisting of 1200 x 1200 pixels and covering xy dimensions of 375 x 375 µm.

Supplementary Table S3. List of primary antibodies for cIEF immunoassays and Western blots

Proteins	Full names	Function	Cat. No.	Vendors
Akt	Protein kinase B	Protein kinase	8312	Santa Cruz Biotech (Dallas, TX)
p-Akt (Thr308)	Protein kinase B, phosphor-isoform	Protein kinase	13038	Cell Signaling (Danvers, MA)
p-Akt (Ser473)	Protein kinase B, phosphor-isoform	Protein kinase	4060	Cell Signaling
p-Akt (Ser477)	Protein kinase B, phosphor-isoform	Protein kinase	Custom antibodies*	Genscript (Piscataway, NJ)
4EBP1	Eukaryotic translation initiation factor 4E-binding protein	Translation repressor	9644	Cell Signaling
p-4EBP1 (Thr37/46)	Eukaryotic translation initiation factor 4E-binding protein, phosphor-isoform	Translation repressor	2855	Cell Signaling
p-4EBP1 (Thr70)	Eukaryotic translation initiation factor 4E-binding protein, phosphor-isoform	Translation repressor	9455	Cell Signaling
BID	BH3 interacting-domain death agonist	Death agonist/activator of Bax	2002	Cell Signaling
HMGCS2	3-hydroxy-3-methylglutaryl-CoA synthase 2 (mitochondrial)	Ketogenesis, cholesterol biosynthesis	137043	Abcam
FABP1	Liver-specific fatty acid binding protein	Fatty acid transport	7847	Abcam
FABP4	Adipocyte and macrophage-specific fatty acid binding protein	Fatty acid transport	92501	Abcam
FABP5	Epidermal fatty acid binding protein	Fatty acid transport	128650	Abcam
LC3A/B	Autophagy marker Light Chain 3A/B	Autophagosome proteins	12741	Cell Signaling

*Primary antibodies against p-Akt (Ser477) was custom-built by Genscript using peptide sequences and protocols described previously by Liu *et al.* Nature 2014;508:541-545.
